# Preoperative nutritional status and its association with postoperative complications and survival in patients undergoing total hip arthroplasty: a retrospective cohort analysis

**DOI:** 10.3389/fpubh.2026.1942939

**Published:** 2026-10-22

**Authors:** Yijun Lian, Zhenyang Yu, Jianing Lu, Junhong Yan, Bowen Han, Yongkang Zhu

**Affiliations:** Department of Orthopedics, Orthopedics Hospital, The First Affiliated Hospital of Henan Medical University, Weihui City, Henan, China

**Keywords:** hip arthroplasty, malnutrition, nutritional screening, postoperative complications, prognostic nutritional index, survival analysis

## Abstract

**Background:**

Adverse preoperative nutritional indicators are common among patients undergoing hip arthroplasty and have been associated with poorer surgical outcomes. However, evidence simultaneously evaluating multiple preoperative nutritional measures in relation to short-term postoperative complications and longer-term survival in patients undergoing total hip arthroplasty remains limited.

**Objective:**

To evaluate the associations of preoperative nutritional status, assessed using the Prognostic Nutritional Index (PNI), serum albumin, and Nutritional Risk Screening 2002 (NRS-2002), with 30-day postoperative complications and overall survival in patients undergoing total hip arthroplasty (THA).

**Methods:**

This single-center retrospective cohort study enrolled 426 consecutive patients who underwent primary THA between January 2020 and December 2023 at a tertiary academic medical center. Patients were stratified into three preoperative PNI strata: PNI ≥ 45 (*n* = 198), PNI 40–44.9 (*n* = 142), and PNI < 40 (*n* = 86). The primary outcomes were 30-day postoperative complications and estimated 3-year overall survival. Logistic regression and Cox proportional hazards models were used to explore adjusted associations.

**Results:**

The overall 30-day complication rate was 18.3% (78/426). Patients with PNI < 40 had a significantly higher complication rate (34.9%) than those with PNI 40–44.9 (19.0%) and PNI ≥ 45 (10.6%; *p* < 0.001). In the reported adjusted model, PNI < 40 (OR = 3.42, 95% CI: 2.01–5.82; *p* < 0.001), serum albumin < 35 g/L (OR = 2.64, 95% CI: 1.58–4.41; *p* < 0.001), and NRS-2002 score ≥ 3 (OR = 2.21, 95% CI: 1.35–3.62; *p* = 0.002) were associated with higher odds of postoperative complications. The estimated 3-year overall survival probabilities were 93.4, 82.4, and 68.6% for the PNI ≥ 45, PNI 40–44.9, and PNI < 40 strata, respectively (log-rank *p* < 0.001). In the reported Cox model, PNI < 40 was associated with higher mortality (HR = 2.96, 95% CI: 1.78–4.92; *p* < 0.001).

**Conclusion:**

Lower preoperative PNI, hypoalbuminemia, and NRS-2002-defined nutritional risk were associated with higher postoperative complication risk and lower estimated long-term survival after THA. These measures may assist preoperative risk stratification. Prospective studies are required to determine whether nutritional interventions modify postoperative complications or survival.

## 1 Introduction

Total hip arthroplasty (THA) is among the most commonly performed orthopedic procedures worldwide, offering significant improvements in pain relief, functional mobility, and quality of life for patients with end-stage hip diseases including osteoarthritis, femoral neck fractures, and avascular necrosis of the femoral head (1, 2). The global demand for THA continues to rise, driven by an aging population and expanding indications, with projections estimating substantial increases in procedure volumes over the coming decades (3). Despite advances in surgical techniques, implant design, and perioperative care protocols, postoperative complications such as surgical site infections (SSIs), venous thromboembolism (VTE), periprosthetic fractures, and medical complications remain clinically significant concerns that contribute to prolonged hospitalization, increased healthcare costs, and impaired patient outcomes (4, 5).

Nutritional status has emerged as an important factor associated with surgical outcomes. Malnutrition is prevalent among surgical populations, particularly in older adults patients undergoing major orthopedic procedures, with reported prevalence rates ranging from 12.5 to 50.6% depending on the screening tool and patient population studied (6, 7). The pathophysiological consequences of malnutrition include impaired immune function, delayed wound healing, decreased muscle mass and strength, and compromised physiological reserves—all of which may predispose patients to adverse perioperative outcomes (8, 9).

Several nutritional assessment tools have been developed and validated in surgical populations. The Prognostic Nutritional Index (PNI), calculated from serum albumin levels and peripheral lymphocyte counts, was originally developed by Onodera et al. as a predictor of postoperative outcomes in gastrointestinal surgery and has since been widely applied across various surgical disciplines (10). The Nutritional Risk Screening 2002 (NRS-2002), endorsed by the European Society for Clinical Nutrition and Metabolism (ESPEN), provides a validated approach for identifying patients at nutritional risk who may benefit from nutritional intervention (11). Serum albumin, while influenced by non-nutritional factors such as inflammation and hepatic function, remains a widely used biomarker that reflects protein-calorie nutritional status and systemic inflammatory burden (12).

In the context of total joint arthroplasty, several studies have demonstrated associations between malnutrition markers and adverse postoperative outcomes. Bohl et al. reported that preoperative hypoalbuminemia independently predicted SSIs, pneumonia, prolonged length of stay (LOS), and readmission after total joint arthroplasty (13). A recent systematic review and meta-analysis confirmed that malnutrition was associated with increased postoperative complications following THA (14). Furthermore, PNI has been evaluated as a prognostic marker for postoperative outcomes in hip fracture populations (15, 16). However, few studies have simultaneously evaluated multiple nutritional indicators and examined their associations with both short-term complications and longer-term survival in primary THA populations encompassing both traumatic and nontraumatic indications.

Therefore, the present study aimed to: (1) characterize preoperative nutritional status in patients undergoing primary THA using PNI, serum albumin, and NRS-2002; (2) determine the association between these preoperative nutritional indicators and 30-day postoperative complication rates; and (3) evaluate their prognostic associations with estimated 3-year overall survival. We hypothesized that lower PNI, hypoalbuminemia, and NRS-2002-defined nutritional risk would be associated with increased postoperative complications and reduced overall survival.

## 2 Methods

### 2.1 Study design and patient population

This retrospective cohort study was conducted at the Orthopedics Hospital, The First Affiliated Hospital of Henan Medical University, in accordance with the Declaration of Helsinki. The requirement for informed consent was waived due to the retrospective nature of the study.

We consecutively enrolled all patients aged 18 years or older who underwent primary unilateral THA between January 1, 2020, and December 31, 2023. Exclusion criteria included: (1) revision arthroplasty; (2) bilateral simultaneous THA; (3) pathological fractures secondary to malignancy; (4) pre-existing active infection at the surgical site; (5) incomplete preoperative laboratory data necessary for nutritional assessment; (6) loss to follow-up within 30 days postoperatively; and (7) concurrent immunosuppressive therapy or active autoimmune disease. A total of 512 patients were initially screened, and after applying exclusion criteria, 426 patients were included in the final analysis.

### 2.2 Nutritional assessment

Preoperative nutritional status was assessed within 48 h before surgery using the following three nutritional indicators or assessment approaches:

Prognostic nutritional index (PNI): Calculated as 10 × serum albumin (g/dL) + 0.005 × total lymphocyte count (/mm^3^). For analysis, patients were grouped into three PNI strata: PNI ≥ 45, PNI 40–44.9, and PNI < 40. These thresholds were adopted from the original Onodera prognostic risk ranges and are used here for stratification rather than as formal diagnostic categories of malnutrition (10).

Serum albumin: measured by the bromocresol green method. Hypoalbuminemia was defined as serum albumin < 35 g/L, consistent with established clinical thresholds (13).

Nutritional risk screening 2002 (NRS-2002): a validated screening tool incorporating nutritional status, disease severity, and age. A total score ≥ 3 indicated nutritional risk and the need for further nutritional assessment (11).

### 2.3 Data collection

Clinical data were extracted from the hospital electronic medical record system by two independent investigators. Demographic variables included age, sex, body mass index (BMI), and smoking status. Comorbidities were recorded and quantified using the Charlson Comorbidity Index (CCI), including hypertension, diabetes mellitus, cardiovascular disease, chronic kidney disease, chronic obstructive pulmonary disease, and cerebrovascular disease. Surgical variables included the American Society of Anesthesiologists (ASA) classification, surgical approach (anterolateral, posterolateral, or direct anterior), anesthesia type, operative time, estimated intraoperative blood loss, and indication for surgery (osteoarthritis, femoral neck fracture, avascular necrosis, or other). Surgical indication was considered an important source of clinical heterogeneity because fracture-related and nontraumatic indications may differ in urgency, baseline health status, inflammatory burden, and mortality risk. Preoperative laboratory parameters included complete blood count, serum albumin, prealbumin, total protein, C-reactive protein (CRP), and erythrocyte sedimentation rate (ESR).

### 2.4 Outcome measures

The primary outcome was the incidence of any postoperative complication within 30 days of surgery. Complications were categorized as surgical (surgical site infection, periprosthetic fracture, implant-related complications, wound dehiscence, hematoma requiring drainage, and dislocation) or medical (pneumonia, urinary tract infection, venous thromboembolism including deep vein thrombosis and pulmonary embolism, acute kidney injury, cardiac events, cerebrovascular events, and delirium). Secondary outcomes included individual complication types, length of hospital stay, 90-day unplanned readmission rate, and estimated 3-year overall survival. Survival data were obtained through medical record review and telephone follow-up, with the last follow-up date being December 31, 2024. In time-to-event analyses, patients alive at their last documented follow-up were treated as censored observations.

### 2.5 Statistical analysis

Continuous variables were expressed as mean ± standard deviation (SD) or median (interquartile range, IQR) depending on the distribution, assessed by the Shapiro–Wilk test. Categorical variables were presented as frequencies and percentages. Comparisons among the three PNI strata were performed using one-way analysis of variance (ANOVA) or the Kruskal-Wallis test for continuous variables and the chi-square test or Fisher exact test for categorical variables. *Post hoc* pairwise comparisons were conducted using the Bonferroni correction.

Receiver operating characteristic (ROC) curve analysis was performed to evaluate the discriminative ability of PNI, serum albumin, and NRS-2002 for 30-day postoperative complications. The area under the curve (AUC) with 95% confidence intervals (CI) was calculated, and an optimal cutoff was identified using the Youden index. Because the PNI cutoff was derived and evaluated in the same cohort, it was considered exploratory rather than clinically validated. Univariate and multivariable logistic regression analyses were used to examine associations with 30-day postoperative complications, with results expressed as odds ratios (OR) and 95% CI. The multivariable model is presented as an exploratory adjusted analysis. Particular caution was applied to interpretation because PNI directly incorporates serum albumin and lymphocyte count and therefore overlaps mathematically and biologically with albumin and other nutritional, inflammatory, and health-status variables. Coefficients for PNI and albumin were not interpreted as mutually independent nutritional effects. Operative time and intraoperative blood loss were considered perioperative variables occurring after baseline nutritional assessment and were distinguished from baseline factors when interpreting the model. Overall survival was estimated using the Kaplan–Meier method and compared among PNI strata using the log-rank test. Reported 3-year survival probabilities are Kaplan–Meier estimates in the presence of censoring and should not be interpreted as crude 36-month proportions for the entire cohort. Cox proportional hazards regression was used to explore adjusted associations with mortality, with results expressed as hazard ratios (HR) and 95% CI. Given the 78 complication events and 52 deaths, multivariable estimates were interpreted cautiously because of the potential for overfitting. The proportional hazards assumption was verified using Schoenfeld residuals. All statistical analyses were performed using R software version 4.3.1 (R Foundation for Statistical Computing, Vienna, Austria). A two-sided *p* value < 0.05 was considered statistically significant.

## 3 Results

### 3.1 Patient characteristics

A total of 426 patients were included in the final analysis. The mean age was 68.7 ± 11.2 years, and 246 (57.7%) were female. The most common indications for THA were osteoarthritis (42.5%), femoral neck fracture (31.2%), and avascular necrosis (18.3%). Based on preoperative PNI, 198 patients (46.5%) were in the PNI ≥ 45 stratum, 142 (33.3%) were in the PNI 40–44.9 stratum, and 86 (20.2%) were in the PNI < 40 stratum. The baseline demographic and clinical characteristics are presented in Table 1.

**Table 1:** Baseline demographic and clinical characteristics of patients stratified by preoperative PNI.

Variable	Total (*n* = 426)	PNI ≥ 45 (*n* = 198)	PNI 40–44.9 (*n* = 142)	PNI < 40 (*n* = 86)	*p* value
Age (years), mean ± SD	68.7 ± 11.2	64.2 ± 10.5	70.8 ± 10.1	75.6 ± 9.8	< 0.001
Female, *n* (%)	246 (57.7)	105 (53.0)	84 (59.2)	57 (66.3)	0.093
BMI (kg/m^2^), mean ± SD	24.2 ± 3.8	25.3 ± 3.4	23.8 ± 3.6	22.1 ± 4.2	< 0.001
BMI < 18.5, *n* (%)	38 (8.9)	8 (4.0)	12 (8.5)	18 (20.9)	< 0.001
Current smoker, *n* (%)	72 (16.9)	28 (14.1)	24 (16.9)	20 (23.3)	0.158
Hypertension, *n* (%)	198 (46.5)	82 (41.4)	68 (47.9)	48 (55.8)	0.065
Diabetes mellitus, *n* (%)	96 (22.5)	32 (16.2)	34 (23.9)	30 (34.9)	0.002
Cardiovascular disease, *n* (%)	78 (18.3)	26 (13.1)	28 (19.7)	24 (27.9)	0.009
CCI, median (IQR)	2 (1–3)	1 (0–2)	2 (1–3)	3 (2–4)	< 0.001
ASA III–IV, *n* (%)	118 (27.7)	36 (18.2)	42 (29.6)	40 (46.5)	< 0.001
Osteoarthritis, *n* (%)	181 (42.5)	98 (49.5)	56 (39.4)	27 (31.4)	0.011
Femoral neck fracture, *n* (%)	133 (31.2)	46 (23.2)	50 (35.2)	37 (43.0)	0.002
Avascular necrosis, *n* (%)	78 (18.3)	38 (19.2)	26 (18.3)	14 (16.3)	0.846
Other indications, *n* (%)	34 (8.0)	16 (8.1)	10 (7.0)	8 (9.3)	0.822
Operative time (min), mean ± SD	98.3 ± 28.6	92.1 ± 24.3	100.7 ± 29.8	108.5 ± 32.1	< 0.001
Blood loss (mL), median (IQR)	350 (200–500)	300 (180–420)	380 (220–520)	420 (280–600)	< 0.001

Patients in the lower PNI strata were significantly older, had lower BMI, higher CCI scores, higher ASA grades, and a greater proportion of femoral neck fracture indications than those in the PNI ≥ 45 stratum (all *p* < 0.05). Patients with PNI < 40 also had longer operative times and greater intraoperative blood loss.

### 3.2 Preoperative nutritional parameters

Detailed preoperative nutritional and laboratory parameters are shown in Table 2. Statistically significant differences were observed across the three PNI strata for serum albumin, prealbumin, total protein, hemoglobin, total lymphocyte count, PNI, and NRS-2002 scores (all *p* < 0.001). Inflammatory markers, including CRP and ESR, were also higher in the lower PNI strata.

**Table 2:** Preoperative nutritional and laboratory parameters by PNI stratum.

Parameter	Total (*n* = 426)	PNI ≥ 45 (*n* = 198)	PNI 40–44.9 (*n* = 142)	PNI < 40 (*n* = 86)	*p* value
Albumin (g/L), mean ± SD	37.6 ± 5.2	41.2 ± 3.1	36.4 ± 3.8	31.5 ± 4.6	< 0.001
Albumin < 35 g/L, *n* (%)	124 (29.1)	12 (6.1)	42 (29.6)	70 (81.4)	< 0.001
Prealbumin (mg/L), mean ± SD	222.1 ± 62.6	256.3 ± 48.2	208.4 ± 52.6	165.8 ± 58.3	< 0.001
Total protein (g/L), mean ± SD	64.3 ± 7.9	67.8 ± 6.1	63.1 ± 7.2	58.3 ± 8.4	< 0.001
Hemoglobin (g/L), mean ± SD	118.8 ± 19.0	128.3 ± 14.2	115.4 ± 16.8	102.7 ± 19.6	< 0.001
Hb < 100 g/L, *n* (%)	68 (16.0)	10 (5.1)	24 (16.9)	34 (39.5)	< 0.001
TLC (×10^9^/L), mean ± SD	1.58 ± 0.50	1.86 ± 0.42	1.48 ± 0.38	1.12 ± 0.46	< 0.001
PNI, mean ± SD	44.5 ± 5.9	49.6 ± 3.4	42.5 ± 1.4	36.2 ± 3.1	< 0.001
NRS-2002, median (IQR)	2 (1–3)	1 (0–2)	2 (1–3)	4 (3–5)	< 0.001
NRS-2002 ≥ 3, *n* (%)	148 (34.7)	28 (14.1)	52 (36.6)	68 (79.1)	< 0.001
CRP (mg/L), median (IQR)	5.8 (2.1–14.6)	3.2 (1.2–6.8)	6.4 (2.8–15.2)	12.8 (5.6–28.4)	< 0.001
ESR (mm/h), mean ± SD	22.1 ± 16.2	15.6 ± 10.2	24.8 ± 15.4	32.6 ± 21.2	< 0.001

### 3.3 Postoperative complications

The overall 30-day postoperative complication rate was 18.3% (78/426). The complication rates differed significantly among the three PNI strata: 10.6% (21/198) for PNI ≥ 45, 19.0% (27/142) for PNI 40–44.9, and 34.9% (30/86) for PNI < 40 (*p* < 0.001). A detailed comparison of individual complication types is presented in Table 3.

**Table 3:** Comparison of 30-day postoperative complications by preoperative PNI stratum.

Complication	Total (*n* = 426)	PNI ≥ 45 (*n* = 198)	PNI 40–44.9 (*n* = 142)	PNI < 40 (*n* = 86)	*p* value
Any complication, *n* (%)	78 (18.3)	21 (10.6)	27 (19.0)	30 (34.9)	< 0.001
Surgical complications					
SSI (superficial)	18 (4.2)	4 (2.0)	6 (4.2)	8 (9.3)	0.014
SSI (deep/organ-space)	8 (1.9)	1 (0.5)	2 (1.4)	5 (5.8)	0.008
Wound dehiscence	12 (2.8)	2 (1.0)	4 (2.8)	6 (7.0)	0.015
Periprosthetic fracture	6 (1.4)	2 (1.0)	2 (1.4)	2 (2.3)	0.672
Dislocation	10 (2.3)	3 (1.5)	3 (2.1)	4 (4.7)	0.254
Hematoma	8 (1.9)	2 (1.0)	3 (2.1)	3 (3.5)	0.363
Medical complications					
Pneumonia	16 (3.8)	3 (1.5)	6 (4.2)	7 (8.1)	0.016
UTI	14 (3.3)	3 (1.5)	5 (3.5)	6 (7.0)	0.048
DVT	15 (3.5)	4 (2.0)	5 (3.5)	6 (7.0)	0.093
Pulmonary embolism	4 (0.9)	1 (0.5)	1 (0.7)	2 (2.3)	0.296
Acute kidney injury	8 (1.9)	1 (0.5)	3 (2.1)	4 (4.7)	0.046
Cardiac events	6 (1.4)	1 (0.5)	2 (1.4)	3 (3.5)	0.131
Delirium	12 (2.8)	2 (1.0)	4 (2.8)	6 (7.0)	0.015
LOS (days), median (IQR)	8 (6–11)	6 (5–8)	8 (6–11)	12 (9–16)	< 0.001
90-day readmission, *n* (%)	34 (8.0)	8 (4.0)	12 (8.5)	14 (16.3)	0.001
30-day mortality, *n* (%)	6 (1.4)	0 (0)	2 (1.4)	4 (4.7)	0.008

Surgical site infections (both superficial and deep), wound dehiscence, pneumonia, urinary tract infections, acute kidney injury, and delirium were reported more frequently in the lower PNI strata. The median length of hospital stay was 12 days in patients with PNI < 40, compared with 8 days for PNI 40–44.9 and 6 days for PNI ≥ 45 (*p* < 0.001). The 90-day unplanned readmission rates were 16.3, 8.5, and 4.0% in the PNI < 40, PNI 40–44.9, and PNI ≥ 45 strata, respectively (*p* = 0.001).

### 3.4 Risk factor analysis for postoperative complications

Univariate logistic regression analysis identified multiple factors associated with 30-day postoperative complications (Table 4). Age ≥ 75 years, BMI < 18.5 kg/m^2^, diabetes mellitus, CCI ≥ 3, ASA III–IV, PNI strata, serum albumin < 35 g/L, NRS-2002 ≥ 3, hemoglobin < 100 g/L, CRP > 10 mg/L, operative time > 120 min, and intraoperative blood loss > 500 mL showed statistically significant associations in the reported analysis.

**Table 4:** Univariate logistic regression analysis of risk factors for 30-day postoperative complications.

Variable	Complication (*n* = 78)	No complication (*n* = 348)	OR (95% CI)	*p* value
Age ≥ 75 years	37 (47.4%)	87 (25.0%)	2.70 (1.64–4.45)	< 0.001
Female sex	47 (60.3%)	199 (57.2%)	1.14 (0.69–1.87)	0.614
BMI < 18.5 kg/m^2^	14 (17.9%)	24 (6.9%)	2.96 (1.45–6.01)	0.003
Current smoker	18 (23.1%)	54 (15.5%)	1.63 (0.90–2.97)	0.110
Diabetes mellitus	27 (34.6%)	69 (19.8%)	2.15 (1.27–3.65)	0.004
Cardiovascular disease	22 (28.2%)	56 (16.1%)	2.05 (1.16–3.61)	0.013
CCI ≥ 3	33 (42.3%)	83 (23.9%)	2.35 (1.41–3.90)	0.001
ASA III–IV	37 (47.4%)	81 (23.3%)	2.98 (1.80–4.92)	< 0.001
PNI < 40 (vs. ≥ 45)	30 (38.5%)	56 (16.1%)	4.52 (2.40–8.51)	< 0.001
PNI 40–44.9 (vs. ≥ 45)	27 (34.6%)	115 (33.0%)	1.98 (1.07–3.67)	0.030
Albumin < 35 g/L	41 (52.6%)	83 (23.9%)	3.55 (2.15–5.86)	< 0.001
NRS-2002 ≥ 3	45 (57.7%)	103 (29.6%)	3.24 (1.97–5.33)	< 0.001
Hemoglobin < 100 g/L	22 (28.2%)	46 (13.2%)	2.58 (1.45–4.60)	0.001
CRP > 10 mg/L	33 (42.3%)	79 (22.7%)	2.50 (1.50–4.17)	< 0.001
Operative time > 120 min	23 (29.5%)	63 (18.1%)	1.89 (1.09–3.27)	0.023
Blood loss > 500 mL	25 (32.1%)	65 (18.7%)	2.05 (1.20–3.50)	0.009

Table 5 presents the reported exploratory multivariable logistic regression model. Statistically significant adjusted associations with 30-day postoperative complications were reported for PNI < 40 and PNI 40–44.9 (each versus PNI ≥ 45), serum albumin < 35 g/L, NRS-2002 score ≥ 3, age ≥ 75 years, ASA III–IV, diabetes mellitus, BMI < 18.5 kg/m^2^, hemoglobin < 100 g/L, CCI ≥ 3, and blood loss > 500 mL; operative time > 120 min did not reach statistical significance. Because of the mathematical and biological overlap among nutritional and health-status variables and the limited number of complication events, these adjusted estimates should be interpreted as exploratory associations rather than mutually independent effects.

**Table 5:** Exploratory multivariable logistic regression analysis for 30-day postoperative complications.

Variable	Adjusted OR	95% CI	*p* value
PNI < 40 (vs. ≥ 45)	3.42	2.01–5.82	< 0.001
PNI 40–44.9 (vs. ≥ 45)	1.87	1.12–3.12	0.016
Serum albumin < 35 g/L	2.64	1.58–4.41	< 0.001
NRS-2002 score ≥ 3	2.21	1.35–3.62	0.002
Age ≥ 75 years	1.93	1.21–3.08	0.006
ASA grade III–IV	2.48	1.47–4.18	0.001
Diabetes mellitus	1.76	1.08–2.87	0.024
BMI < 18.5 kg/m^2^	2.15	1.18–3.92	0.013
Hemoglobin < 100 g/L	1.89	1.14–3.13	0.014
CCI ≥ 3	1.68	1.02–2.77	0.042
Operative time > 120 min	1.54	0.93–2.55	0.094
Blood loss > 500 mL	1.72	1.05–2.82	0.032

### 3.5 Discriminative performance of nutritional indicators

ROC curve analysis showed that PNI had the highest discriminative ability for 30-day postoperative complications in this cohort, with an AUC of 0.736 (95% CI: 0.696–0.766), followed by serum albumin (AUC = 0.687, 95% CI: 0.637–0.727) and NRS-2002 (AUC = 0.642, 95% CI: 0.592–0.682). The Youden index identified a PNI cutoff of 42.8 (sensitivity 75.0%, specificity 62.0%). Because this cutoff was derived and evaluated in the same dataset, it should be considered exploratory and requires internal or external validation before clinical application. The pairwise comparison of AUCs indicated greater discrimination for PNI than NRS-2002 (*p* = 0.012), whereas the difference between PNI and serum albumin did not reach statistical significance (*p* = 0.068). These results are illustrated in Figure 1.

**Figure 1:**
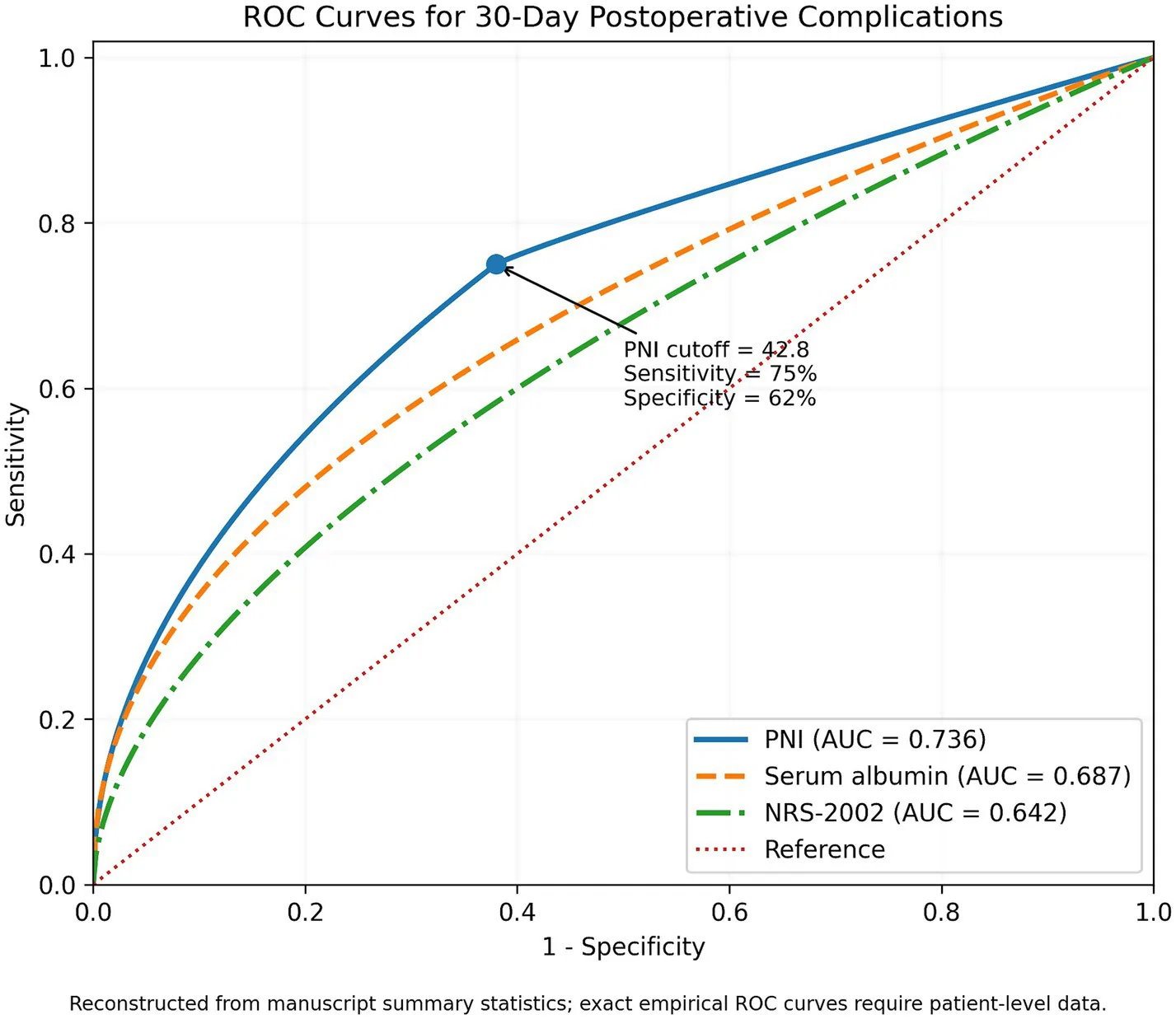
Receiver operating characteristic (ROC) curves for the prognostic nutritional index (PNI), serum albumin, and NRS-2002 in predicting 30-day postoperative complications following total hip arthroplasty. The PNI cutoff identified in this cohort is exploratory and requires validation.

### 3.6 Survival analysis

The median follow-up duration was 28.4 months (IQR: 16.2–36.0 months). During follow-up, 52 patients (12.2%) died. The estimated 3-year overall survival probabilities were 93.4% (95% CI: 89.7–97.1%) for PNI ≥ 45, 82.4% (95% CI: 76.1–88.7%) for PNI 40–44.9, and 68.6% (95% CI: 58.8–78.4%) for PNI < 40. The log-rank test indicated a difference among the three PNI strata (χ^2^ = 18.74, *p* < 0.001). Because enrollment continued through December 2023 whereas follow-up ended on December 31, 2024, not all participants could have reached 36 months of follow-up; therefore, the 3-year values are Kaplan–Meier estimates based on censored data and should be interpreted in light of the number remaining at risk at 36 months. Kaplan–Meier survival curves are presented in Figure 2.

**Figure 2:**
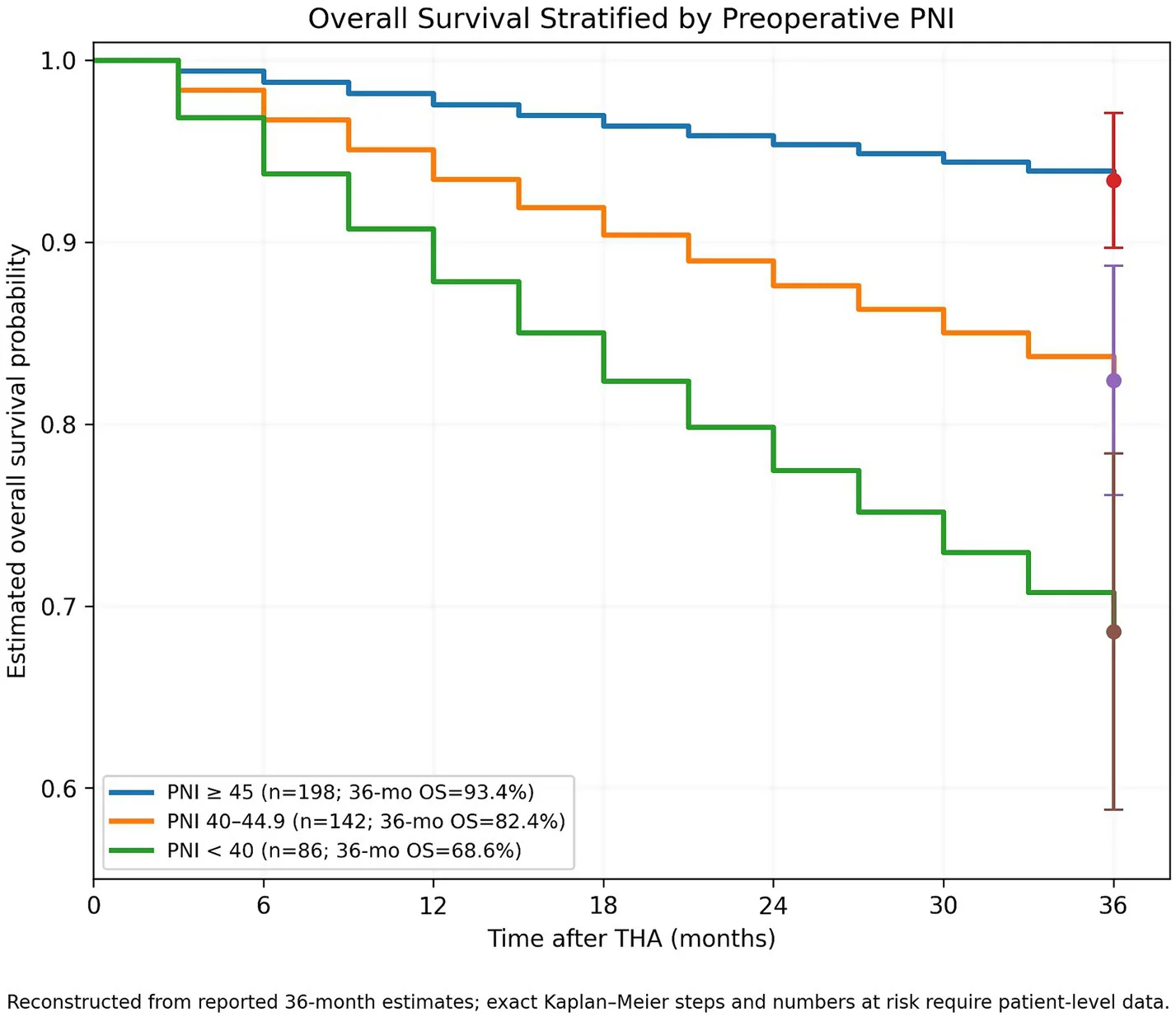
Kaplan–Meier survival curves for estimated 3-year overall survival stratified by preoperative PNI.

### 3.7 Cox proportional hazards regression for overall survival

The reported exploratory multivariable Cox model showed statistically significant associations with mortality for PNI < 40 (HR = 2.96, 95% CI: 1.78–4.92; *p* < 0.001), PNI 40–44.9 (HR = 1.74, 95% CI: 1.06–2.85; *p* = 0.028), serum albumin < 35 g/L (HR = 2.38, 95% CI: 1.42–3.99; *p* = 0.001), NRS-2002 score ≥ 3 (HR = 1.92, 95% CI: 1.18–3.12; *p* = 0.009), age ≥ 75 years (HR = 2.15, 95% CI: 1.36–3.40; *p* = 0.001), ASA III–IV (HR = 2.31, 95% CI: 1.38–3.87; *p* = 0.001), BMI < 18.5 kg/m^2^ (HR = 1.96, 95% CI: 1.08–3.56; *p* = 0.027), hemoglobin < 100 g/L (HR = 1.72, 95% CI: 1.04–2.84; *p* = 0.035), and CCI ≥ 3 (HR = 1.84, 95% CI: 1.12–3.02; *p* = 0.016) (Table 6). Given the limited number of deaths and overlap among several predictors, these estimates should be interpreted cautiously. The forest plot is displayed in Figure 3.

**Table 6:** Exploratory multivariable Cox proportional hazards analysis of overall survival.

Variable	Adjusted HR	95% CI	*p* value
PNI < 40 (vs. ≥ 45)	2.96	1.78–4.92	< 0.001
PNI 40–44.9 (vs. ≥ 45)	1.74	1.06–2.85	0.028
Serum albumin < 35 g/L	2.38	1.42–3.99	0.001
NRS-2002 score ≥ 3	1.92	1.18–3.12	0.009
Age ≥ 75 years	2.15	1.36–3.40	0.001
ASA grade III–IV	2.31	1.38–3.87	0.001
Diabetes mellitus	1.58	0.97–2.57	0.066
BMI < 18.5 kg/m^2^	1.96	1.08–3.56	0.027
Hemoglobin < 100 g/L	1.72	1.04–2.84	0.035
CCI ≥ 3	1.84	1.12–3.02	0.016
Operative time > 120 min	1.41	0.85–2.34	0.184
Blood loss > 500 mL	1.63	0.99–2.68	0.054

**Figure 3:**
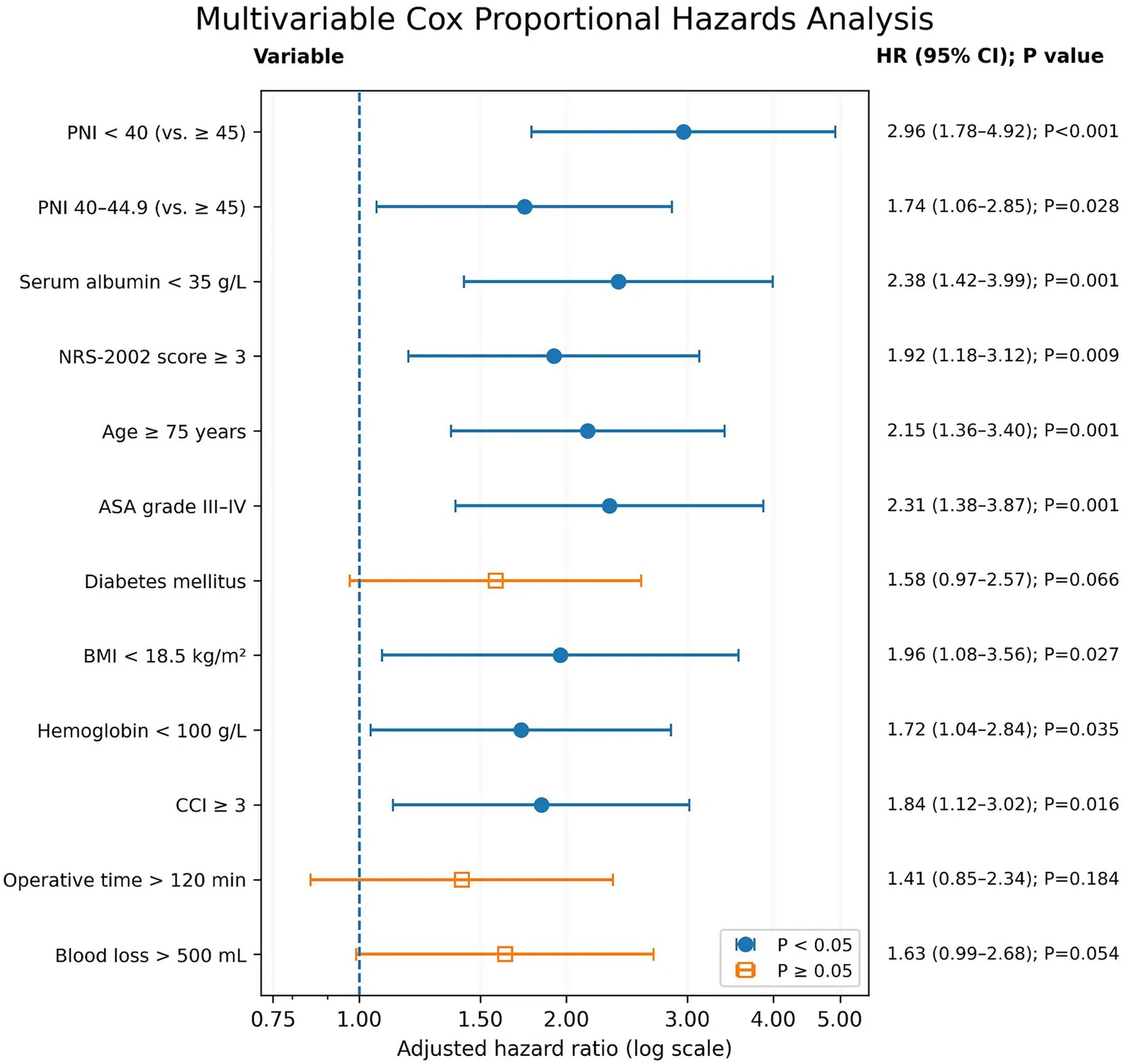
Forest plot of the exploratory multivariable Cox proportional hazards analysis of overall survival following total hip arthroplasty. Blue circles indicate statistically significant associations (*p* < 0.05); orange squares indicate non-significant associations.

Within the reported Cox model, PNI < 40 had the largest hazard ratio among the listed predictors (HR = 2.96, 95% CI: 1.78–4.92; *p* < 0.001). Serum albumin < 35 g/L (HR = 2.38, 95% CI: 1.42–3.99; *p* = 0.001) and ASA III–IV (HR = 2.31, 95% CI: 1.38–3.87; *p* = 0.001) were also associated with higher mortality. Diabetes mellitus (HR = 1.58, *p* = 0.066), operative time > 120 min (HR = 1.41, *p* = 0.184), and intraoperative blood loss > 500 mL (HR = 1.63, *p* = 0.054) did not meet the prespecified *p* < 0.05 threshold for statistical significance in the survival model.

## 4 Discussion

This retrospective cohort study found that preoperative nutritional indicators were associated with both short-term postoperative complications and longer-term survival outcomes in patients undergoing primary THA. Patients with PNI < 40 had higher odds of 30-day postoperative complications in the reported adjusted model (OR = 3.42) and lower estimated 3-year overall survival than patients with PNI ≥ 45. These findings support the potential value of preoperative nutritional assessment for risk stratification, but they do not establish that nutritional status causally determines postoperative outcomes or that nutritional intervention would improve them.

The overall 30-day complication rate was 18.3%, and complication rates increased across decreasing PNI strata from 10.6% for PNI ≥ 45 to 34.9% for PNI < 40. This graded pattern supports an association between lower PNI and adverse postoperative outcomes but does not establish causality. Our findings are consistent with the systematic review and meta-analysis by Aziz et al., which reported associations between malnutrition and adverse perioperative outcomes after THA (14).

PNI showed the highest discriminative performance among the three nutritional measures evaluated in this cohort (AUC = 0.736), compared with serum albumin (AUC = 0.687) and NRS-2002 (AUC = 0.642). Because PNI incorporates both serum albumin and lymphocyte count, however, its coefficient should not be interpreted as statistically independent of albumin when both are entered in the same model. Chen et al. reported PNI as an independent predictor of 3-year postoperative mortality in older adults with hip fractures patients (15). A related prospective cohort study by Chen et al. also reported an association between PNI and functional outcome after hip fracture (16). Wilson et al. reported that combined malnutrition and frailty was associated with higher complication and mortality risk in patients undergoing elective THA (17). Wu et al. reported higher 1-year mortality among patients with lower GNRI and PNI values, although low GNRI rather than PNI was retained as an independent mortality predictor in their multivariable analysis (18). The internally derived PNI cutoff of 42.8 in the present study should therefore be considered exploratory until validated in an independent dataset.

Hypoalbuminemia (serum albumin < 35 g/L) was associated with complications and mortality in the reported adjusted models, consistent with prior literature. A systematic review and meta-analysis by Yue et al. found that preoperative hypoalbuminemia was associated with higher risks of multiple complications, transfusion, unplanned reoperation, and mortality after primary THA or total knee arthroplasty (19). Kiritsis et al. reported that hypoalbuminemia was associated with higher risks of adverse events, surgical site infection, periprosthetic fracture, and early repeat revision after revision THA (20). Proposed mechanisms linking poor nutritional or inflammatory status with adverse outcomes include impaired wound healing and immune function, although serum albumin is also influenced by inflammation and other non-nutritional factors (21).

NRS-2002 showed lower discrimination than PNI in the ROC analysis but remained associated with postoperative complications in the reported adjusted model. This tool incorporates nutritional status, disease severity, and age and is widely used for nutritional risk screening in hospitalized patients. Shang et al. reported that NRS-2002-defined nutritional risk was associated with postoperative complications and resource utilization among 1,532 patients undergoing total joint arthroplasty (22).

The survival analysis showed marked differences in estimated 3-year overall survival across the PNI strata. These estimates require cautious interpretation because the cohort included heterogeneous surgical indications, including femoral neck fracture in 31.2% of patients. Fracture-related THA may differ from nontraumatic THA in urgency, baseline health status, inflammatory burden, and mortality risk, creating potential residual confounding. In the reported Cox model, age ≥ 75 years, ASA III–IV, CCI ≥ 3, and several nutritional or hematologic variables were associated with mortality, whereas diabetes mellitus did not reach statistical significance (HR = 1.58, *p* = 0.066). Nutritional indices may therefore reflect broader physiologic vulnerability in addition to nutritional status itself (23).

The findings have potential implications for preoperative risk assessment rather than direct evidence for treatment efficacy. PNI can be calculated from routinely available laboratory parameters and may complement, but should not replace, established nutritional screening and clinical assessment. For patients with confirmed nutritional risk, optimization may be considered according to the clinical context and applicable nutritional guidance; however, the present study did not test whether nutritional intervention reduces complications or improves survival. Evidence from THA intervention studies remains limited: for example, Ninomiya et al. evaluated perioperative nutritional supplementation combined with exercise in frail older women undergoing fast-track THA and found selective improvements in muscle strength without clear additional benefits in overall functional performance or quality of life (24). Decisions regarding surgical timing should also distinguish urgent fracture-related THA from nonurgent indications.

This study has several limitations. First, the retrospective design is subject to selection bias, residual confounding, and reverse or unmeasured associations; causality cannot be inferred. Second, this was a single-center study, which may limit generalizability. Third, the cohort combined heterogeneous surgical indications, including femoral neck fracture in approximately one-third of patients, and these groups may differ in urgency, baseline health status, inflammatory burden, and mortality risk. The sample size did not permit adequately powered indication-specific analyses, so residual confounding by indication remains possible. Fourth, PNI is a prognostic index rather than a formal diagnostic criterion for malnutrition; the PNI strata used here were based on historical prognostic thresholds, and the ROC-derived cutoff of 42.8 was derived and evaluated in the same cohort without internal or external validation. Fifth, PNI directly incorporates albumin and lymphocyte count and overlaps biologically with albumin, NRS-2002, BMI, hemoglobin, CRP, ASA grade, and CCI. Simultaneous inclusion of several related variables may cause multicollinearity and unstable effect estimates, so the multivariable results should be viewed as exploratory rather than as evidence of mutually independent effects. Sixth, the logistic and Cox models included multiple predictors relative to 78 complications and 52 deaths, increasing the risk of overfitting. Seventh, operative time and intraoperative blood loss occur after baseline nutritional assessment and may lie on the causal pathway; models including these variables may be susceptible to overadjustment or mediator bias. Eighth, patients with incomplete laboratory data required for nutritional assessment were excluded, and variable-specific missingness for other clinical variables should be fully characterized from the source dataset. Ninth, median follow-up was 28.4 months and not all participants had the opportunity to reach 36 months; consequently, 3-year Kaplan–Meier estimates depend on a smaller late risk set and should be interpreted cautiously. Finally, the study did not evaluate the effect of nutritional intervention, and prospective multicenter studies are needed both to validate the prognostic findings and to test whether nutritional optimization improves clinical outcomes.

## 5 Conclusion

Lower preoperative PNI, hypoalbuminemia, and NRS-2002-defined nutritional risk were associated with higher 30-day postoperative complication risk and lower estimated longer-term survival in patients undergoing primary THA. PNI showed the highest discrimination among the three nutritional measures evaluated in this cohort, but its internally derived cutoff requires validation and its overlap with serum albumin should be considered when interpreting multivariable models. These findings support the use of preoperative nutritional assessment for risk stratification; prospective interventional studies are needed to determine whether nutritional optimization improves postoperative complications or survival.

## Data Availability

The data supporting the findings of this study are available from the corresponding author upon reasonable request, subject to institutional approval and applicable privacy requirements.
